# Environmental Factors Affecting Microbiota Dynamics during Traditional Solid-state Fermentation of Chinese Daqu Starter

**DOI:** 10.3389/fmicb.2016.01237

**Published:** 2016-08-04

**Authors:** Pan Li, Weifeng Lin, Xiong Liu, Xiaowen Wang, Lixin Luo

**Affiliations:** ^1^Guangdong Key Laboratory of Fermentation and Enzyme Engineering, School of Bioscience and Bioengineering, South China University of TechnologyGuangzhou, China; ^2^College of Light Industry and Food Sciences, South China University of TechnologyGuangzhou, China

**Keywords:** solid-state fermentation, Daqu, microbiota dynamics, environmental factors, temperature, relationship

## Abstract

In this study, we investigated the microbiota dynamics during two industrial-scale traditional solid-state fermentation (SSF) processes of Daqu starters. Similar evolution profiles of environmental parameters, enzymatic activities, microbial amounts, and communities were observed during the medium temperature SSF (MTSSF) and low temperature SSF (LTSSF) processes. Orders of *Rickettsiales* and *Streptophyta* only dominated the initial 2 days, and *Eurotiales* only predominated from days 10 to 24, however, phylotypes of *Enterobacteriales, Lactobacillales, Bacillales, Saccharomycetales*, and *Mucorales* both prevailed throughout the MTSSF and LTSSF processes. Nevertheless, the pH in MTSSF process on day 5 were 5.28, while in LTSSF process (4.87) significantly lower (*P* < 0.05). The glucoamylase activities in MTSSF process dropped from 902.71 to 394.33 mg glucose g^-1^ h^-1^ on days 5 to 24, while significantly lower (*P* < 0.05) in LTSSF process and decreased from 512.25 to 268.69 mg glucose g^-1^ h^-1^. The relative abundance of *Enterobacteriales* and *Lactobacillales* in MTSSF process constituted from 10.30 to 71.73% and 2.34 to 16.68%, while in LTSSF process ranged from 3.16 to 41.06% and 8.43 to 57.39%, respectively. The relative abundance of *Eurotiales* in MTSSF process on days 10 to 24 decreased from 36.10 to 28.63%, while obviously higher in LTSSF process and increased from 52.00 to 72.97%. Furthermore, lower bacterial richness but higher fungal richness were displayed, markedly differences in bacterial communities but highly similarities in fungal communities were exhibited, during MTSSF process comparatively to the LTSSF process. Canonical correspondence analysis revealed microbial structure transition happened at thermophilic stages under environmental stress of moisture, pH, acidity, and pile temperature. These profound understanding might help to effectively control the traditional Daqu SSF process by adjusting relevant environmental parameters.

## Introduction

Highly complex microbial communities play a critical role for ecosystems by supporting the main global biogeochemical cycles, however, microorganisms are greatly affected by environmental factors. Over recent decades, extensive studies were performed to provide profound insights into the intricate relationship between the environmental parameters and the microbial dynamics in various ecosystems including marine ecosystems (Gilbert et al., 2012; Tinta et al., 2015), hot springs (Song et al., 2013; Coman et al., 2015), soils (De Gannes et al., 2015; Žifčáková et al., 2016) and composting systems (Lu et al., 2015). These studies have shown that environmental conditions dictate the structuring of microbial composition. Among these environmental factors, temperature was shown to largely affect the microbial dynamics, and the microbial diversity generally decreased with temperature increased (Miller et al., 2009). Consequently, the understanding on how highly variable environmental parameters affect microbial structure might lead to predictable patterns of microbial assemblages.

From an ecological point of view, traditional spontaneous solid-state fermentation (SSF) of cereal starters, which locally called “Daqu” and anciently used as starters to produce Chinese liquor and vinegar, is a dynamic process due to the combined activity of varieties of microbial populations, which were linked to consecutive environmental conditions (Zheng et al., 2012). Various factors, such as temperature, moisture content, pH and acidity, related to one another determine the succession of the different environmental conditions appearing throughout the SSF process. Generally, Daqu is produced in an open-work environment with non-autoclaved raw materials of mixtures of barley, wheat and peas, the preparation process of Daqu mainly involved three stages (Zheng et al., 2012): (i) material grinding, mixing, and shaping; (ii) spontaneous SSF process with temperature controlled; and (iii) drying and ripening. According to the highest inoculation temperature, three typical types of Daqu starters can be distinguished, low temperature Daqu (45–50°C), medium temperature Daqu (50–60°C) and high temperature Daqu (60–65°C). Accordingly, the moisture content obviously decreased from approximately 35–40% to 8–12% during Daqu SSF process (Zheng et al., 2014; Li et al., 2015). In responses to these environmental variations, microbial communities must undergo complex changes during the Daqu SSF process.

Unfortunately, despite a growing understanding of the microbial structure and dynamics during the SSF process of various types of Daqu starters (Nie et al., 2013; Li et al., 2015), however, only two previous studies have addressed the potential links between microbial diversity and environmental factors, moisture content and temperature were found to be strongly correlated with the composition of *Bacillus* sp. and thermophilic fungi (Zheng et al., 2014; Wang and Xu, 2015). Thus, a wide variety of thermophilic and drought-resistant communities, such as *Bacillales, Eurotiales*, and *Mucorales*, have been detected in various types of Daqu starters (Li et al., 2015; Wang and Xu, 2015). Likewise, our recent study also indicated that the composition of the microbial communities was significantly corrected with temperature, acidity and moisture content during Daqu SSF process (Li et al., 2015).

Up to date, the knowledge on how highly variable environmental parameters affect microbial community structure in Daqu SSF process is still scarce, no comparative studies have been performed to clear the relationship of microbial community composition, abundance, and diversity with different environmental factors gradient. In present study, we analyzed the microbiota dynamics and compared the difference in microbial communities during a medium temperature SSF (MTSSF) process and a low temperature SSF (LTSSF) process of Daqu starters by Illumina-based high-throughput sequencing and quantitative PCR (qPCR) analyses, and indicated whether these differences in microbial assemblages were due to environmental factors changes. To our knowledge, this is the first report to perform comparative studies to investigate the influence of environmental factors on microbiota dynamics during Daqu SSF process.

## Materials and Methods

### Solid-state Fermentation of Daqu and Sampling

**FIGURE 1:**
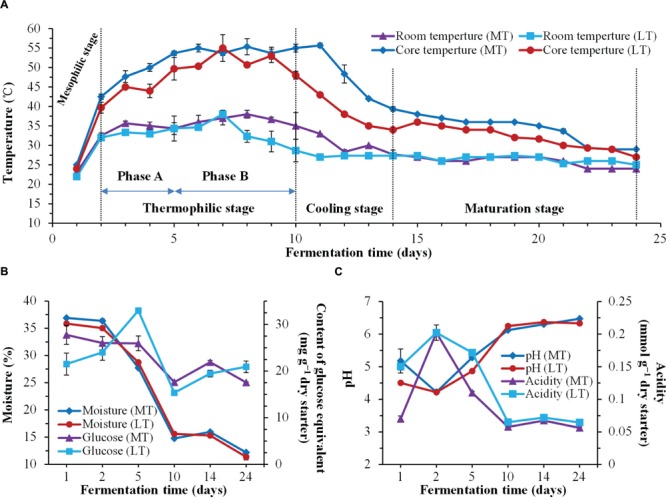
**Dynamics of physicochemical characteristics during the MTSSF and LTSSF processes. (A)** Changes in room temperature and core temperature of Daqu. **(B)** Changes in moisture and reducing sugar (calculated as glucose) contents. **(C)** Changes in pH and acidity.

Spontaneous SSF of Daqu starters at an industrial scale was performed in the fermentation room of a traditional vinegar production factory in Shanxi province, China. Initially, cereal materials of approximately 4200 kg barley and 1800 kg wheat were ground and mixed. Then the mixtures were stirred with the addition of 36–37% water, and were shaped into bricks (28 cm × 18 cm × 5 cm) and layer-by-layer piled in the fermentation rooms. The stacked layers of Daqu blocks were incubated for 24 days with strict temperature control. According to traditional SSF techniques, the variation of room temperature and core temperature of Daqu SSF process was controlled by forced ventilation, and the piles of Daqu blocks were manual turned every 2 days at the thermophilic and cooling stages of SSF process to allow adequate aeration and to decrease the inoculation temperature. To investigate the influence of environmental factors on microbiota dynamics during Daqu SSF process, a MTSSF process and a LTSSF process of Daqu starters were conducted with the highest inoculation temperature reached 48–53°C and 53–55°C (**Figure 1**, Stage B), respectively. The MTSSF and LTSSF processes of Daqu starters were both conducted through two independent experiments. Daqu samples were separately collected at days 1, 2, 5, 10, 14, and 24 according to temperature evolution during the SSF process (**Figure 1**). To obtain adequate information and representation, Daqu blocks from each stage were randomly selected from the upper, middle, and lower locations of two individual processes in triplicates, which were then ground, mixed, and pooled into sterile Stomacher bags (Stomacher Lab System, London, United Kingdom) to provide an experimental Daqu powder sample (approximately 500 g). All of the samples were stored at -20°C for further analysis.

### Physicochemical and Enzymatic Analysis

The room temperature was monitored every day by a thermo-hygrometer (GEMlead TH339, Fuzhou, China) stuck on the wall of fermentation room, and the core temperature of Daqu (pile temperature) was determined every day using a thermograph (Neo Thermo TVS-700, Nippon Avionics, Tokyo, Japan) inserted into Daqu blocks with a depth of 1 cm. The moisture of Daqu was determined by dry/wet weight measurement method at 105°C. The pH was measured with a pH meter (Sartorius PB-10, Germany). The total titratable acidity was determined by titration with 0.02 M NaOH exhibiting a titration endpoint of pH 8.2. The reducing sugar content was determined by DNS method (Miller, 1959). The amino acid nitrogen content and protease activity were determined according to the national professional standard methods (SB/T 10317-1999, 1999; QB/T 4257-2011, 2011). One unit of protease activity was defined as the amount of amino acid nitrogen liberated per hour by 1 g Daqu under the assay conditions. Amylase and glucoamylase activities were determined as our previously described (Li et al., 2015). One unit of amylase activity was defined as the amount of starch liquefied per hour by 1 g Daqu in sodium acetate buffer (50 mM, pH 4.6) at 35°C. One unit of glucoamylase activity was defined as the amount of glucose liberated per hour by 1 g Daqu in sodium acetate buffer (50 mM, pH 4.6) at 40°C.

### DNA Extraction and qPCR Analysis

DNA extraction from Daqu samples was performed using the Soil DNA Kit (Omega Bio-Tek, Norcross, GA, USA) according to the manufacturer’s instructions. qPCR analysis was performed in quadruplicate using the commercial kit (SYBR^®^ Premix Ex TaqTM II, Takara, Dalian, China) with an ABI 7500 Real Time PCR System (Applied Biosystems). Primers pairs P1/P2, Lac1/Lac2, B1/B2, and Y1/Y2 were used to quantify the specific gene abundance of total bacteria, LAB, *Bacillus*, and fungi (Muyzer et al., 1993; Xu et al., 2011), respectively. Each reaction was performed in a 25 μL volume containing 12.5 μL SYBR Premix Ex Taq (Takara, Dalian, China), 0.5 μL of each primer (10 mM) and 2 μL of 10-fold (*Bacillus*) or 100-fold (bacteria, LAB and fungi) dilution DNA template. The qPCR thermocycling steps were as follows: 95°C for 30 s, 40 cycles of 95°C for 5 s, 55°C for 34 s, 72°C for 30 s. The fluorescent products were detected at the annealing step of each cycle. Melting curve analysis and agarose gel electrophoresis were performed to confirm the specificity of the amplification. The amplification efficiency and correlation coefficient (*R*^2^) for the amplification of specific gene of bacteria, *Bacillus*, LAB and fungi were 99.3% and 0.992, 97.1% and 0.990, 94.4% and 0.991, 98.1% and 0.991, respectively.

### Illumina HiSeq Sequencing

The V4 regions of bacterial 16S rRNA gene and ITS1 regions of fungal rRNA genes were amplified used the specific primers 515f/806r (Peiffer et al., 2013) and ITS5-1737F/ITS2-2043R (Huang et al., 2016) with the barcodes, respectively. All PCR reactions were carried out in triplicate 30 μL reactions with 15 μL of Phusion^®^ High-Fidelity PCR Master Mix (New England BioLabs), 0.2 μM of each primer and 10 ng DNA templates. Thermal cycling consisted of initial activation at 98°C for 1 min, followed by 30 cycles of denaturation at 98°C for 10 s, annealing at 50°C for 30 s, and elongation at 72°C for 60 s and finally elongation at 72°C for 5 min. Negative control were treated similarly with the exclusion of template DNA and failed to produce visible PCR products. PCR products were mixed in equimolar ratios and mixture PCR products were purified with QIAquick Gel Extraction Kit (QIAGEN, Dusseldorf, Germany). Sequencing libraries were generated using TruSeq^®^ DNA PCR-Free Sample Preparation Kit (Illumina, USA) following manufacturer’s recommendations and index adaptors were added. The library quality was assessed on the Qubit@ 2.0 Fluorometer (Thermo Scientific) and Agilent Bioanalyzer 2100 system. Finally, the library was sequenced on an Illumina HiSeq2500 platform, at Novogene, Beijing, China.

### Data Processing and Bioinformatics Processing

Paired-end reads from the original DNA fragments were merged using FLASH (Zhang et al., 2014) and assigned to each sample with the unique barcodes. UPARSE software package (Uparse v7.0.1001) with the UPARSE-OTU and UPARSE-OTUref algorithms was used to pick operational taxonomic units (OTUs) at the 97% similarity (Edgar, 2013). Representative sequences were picked for each OTU, and RDP classifier (Version 2.2) was used to annotate the taxonomic information for each representative sequence. Alpha diversity indices Chao1, Shannon, Simpson, ACE and Goods coverage were performed in QIIME (Version 1.7.0) to reflect the diversity and richness of microbial community in different samples (Caporaso et al., 2010). For beta diversity, QIIME calculated both the unweighted and weighted UniFrac distances (Lozupone et al., 2011). Sequencing results are available through the GenBank sequence read archive database under accession number PRJNA316566.

### Statistical Analysis

The statistical significance (*P* ≤ 0.05) of the difference among different batches were identified using a one-way analysis of variance (ANOVA). Pearson’s test was performed to reveal the correlations between environmental variables and abundant classes using SPSS Statistics 19.0. Principal coordinate analysis (PCoA) was performed with the weighted UniFrac distance. Paired *t*-test and wilcoxon tests within the stats R package (Version 2.15.3) were performed to test whether there was a significant difference in the alpha and beta diversity indices among the two batches. In addition, Analysis of similarities (ANOSIM) (Clarke, 1993) and multi-response permutation procedure (MRPP) (He et al., 2010) analyses were further employed to examine the community difference among the two batches. Canonical correspondence analysis (CCA) between Daqu microbial community and measured variables was performed with Canoco 5.0 software.

## Results

### Dynamics of Physicochemical Characteristics

Temperature was a universal indicator used to monitor and control the Daqu SSF process. Dynamics of this parameter and other physicochemical characteristics throughout the SSF process are shown in **Figure 1**. The thermal profile was followed the typical evolution of Daqu SSF process, and allowed to distinguish four stages during the MTSSF and LTSSF processes: the mesophilic stage (days 1 to 2), the thermophilic stage (days 2 to 10), the cooling stage (days 10 to 14), and the maturation stage (days 14 to 24) (**Figure 1A**). However, the temperature of MTSSF process was generally 3–7°C and 5–12°C higher than that in LTSSF process at thermophilic stage and cooling stage (**Figure 1A**), respectively. The temperature increased from 48 to 53°C and 44 to 50°C (Phase A of thermophilic stage), and maintained around 55°C and 50°C (Phase B of thermophilic stage) at thermophilic stage of MTSSF and LTSSF processes, respectively.

Generally, the evolution profiles of other physicochemical parameters were similar during the MTSSF and LTSSF processes. The moisture slightly declined, and then markedly decreased after 2 days and finally fell to 12.18 and 11.38% (**Figure 1B**), respectively. The reducing sugar contents shown a substantially decreased tendency during the MTSSF process with the exception of cooling stage, however, a noteworthy increase of reducing sugar contents (32.94 mg g^-1^ dry starter) could be observed at phase A and followed by a quick depletion (dropped to 15.33 mg g^-1^ dry starter) at phase B of the thermophilic stage during the LTSSF process (**Figure 1B**). Despite the variation in initial pH and titratable acidity, the pH firstly decreased to about 4.22 and the titratable acidity increased to approximately 0.20 mmol g^-1^ at mesophilic stage, but afterward the pH obviously increased and the titratable acidity strongly decreased at the thermophilic stage, and finally the pH and titratable acidity both maintained near 6.40 and 0.06 mmol g^-1^ until the end of MTSSF and LTSSF processes (**Figure 1C**). Nevertheless, significantly (*P* < 0.05) higher in pH and lower in titratable acidity were displayed at the mesophilic and thermophilic stages of the MTSSF process compare with the LTSSF process (**Figure 1C**). The pH and titratable acidity on day 5 were 5.28 and 4.87, 0.11, and 0.17 mmol g^-1^ during MTSSF and LTSSF processes, respectively.

### Dynamics of Enzymatic Activities

**FIGURE 2:**
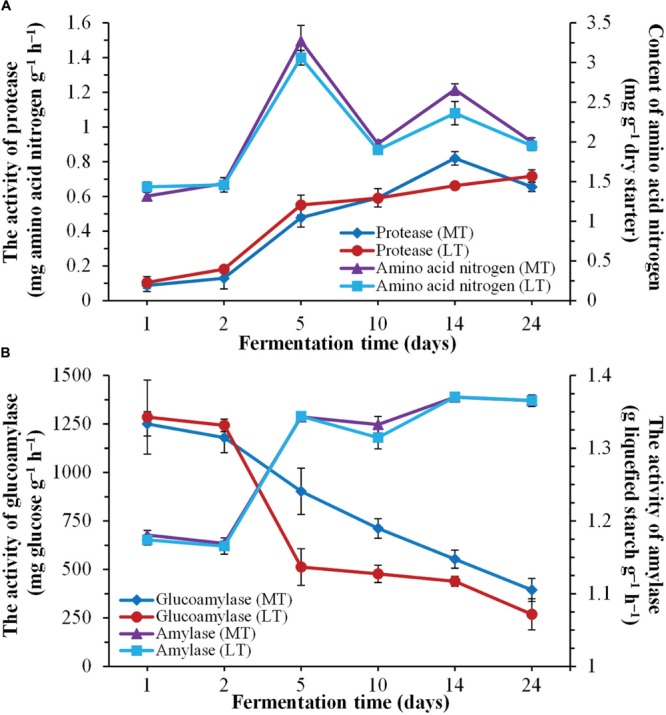
**Dynamics of enzymatic activities during the during the MTSSF and LTSSF processes. (A)** Changes in protease activity and amino acid nitrogen content. **(B)** Changes in glucoamylase and amylase activities.

Dynamics of enzymatic activities throughout the SSF process are shown in **Figure 2**. Overall, the evolution profiles of enzymatic activities were similar during the MTSSF and LTSSF processes. The protease activities increased from 0.09 to 0.82 mg amino acid nitrogen g^-1^ h^-1^ on days 1 to 14 and fell to 0.66 mg amino acid nitrogen g^-1^ h^-1^ at the end of MTSS process, however, the protease activities shown a gradual increased tendency from initial 0.11 to 0.72 mg amino acid nitrogen g^-1^ h^-1^ at the end of the LTSS process (**Figure 2A**). The contents of amino acid nitrogen slightly increased within the first 2 days, and significantly (*P* < 0.05) increased from days 2 to 5 and fluctuated around a peak on day 5. Moreover, significantly (*P* < 0.05) higher in protease activity and amino acid nitrogen content were displayed on day 14 during MTSSF process compare with the LTSSF process (**Figure 2A**). The amylase activities slightly declined within the first 2 days, and then increased rapidly from days 2 to 5, and maintained near 1.37 g liquefied starch g^-1^ h^-1^ until the end of MTSSF and LTSSF processes (**Figure 2B**). However, the glucoamylase activity slightly decreased within the first 2 days, and then obviously declined throughout the MTSSF and LTSSF processes (**Figure 2B**). The glucoamylase activities in MTSSF process dropped steadily from 902.71 to 394.33 mg glucose g^-1^ h^-1^ on days 5 to 24, in LTSSF process from 512.25 to 268.69 mg glucose g^-1^ h^-1^. Significantly (*P* < 0.05) higher in glucoamylase activities were noted after the mesophilic stage of the MTSSF process compare with the LTSSF process (**Figure 2B**).

### Biomass Dynamics by qPCR

**FIGURE 3:**
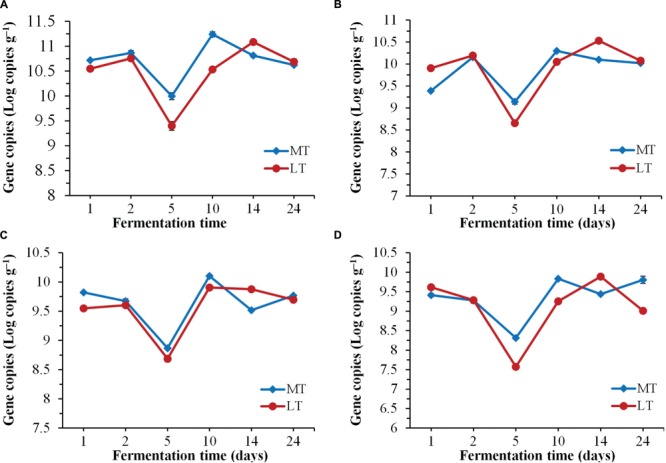
**Dynamics of quantification of total bacteria **(A)**, LAB **(B)**, *Bacillus***(C)**, and fungi **(D)** during the MTSSF and LTSSF processes**.

Dynamics of biomass of total bacteria, LAB, *Bacillus* and fungi by qPCR throughout the SSF process are shown in **Figure 3**. Despite the variation in initial quantities, the levels of total bacteria, LAB, *Bacillus* and fungi firstly decreased significantly (*P* < 0.05) at phase A of thermophilic stages during the SSF process (**Figure 3**). Afterward, their quantities increased significantly (*P* < 0.05) at phase B of thermophilic stages. Notably, higher levels of total bacteria, LAB, *Bacillus* and fungi were displayed at the thermophilic stages of the MTSSF process compare with the LTSSF process. Afterward, the microbial quantity displayed decreased and increased trends at cooling stages during MTSSF and LTSSF processes, respectively. The levels of total bacteria, LAB, *Bacillus* and fungi during the MTSSF and LTSSF processes ranged from 9.40 to 11.24 Log copies g^-1^, 8.6 to 10.30 Log copies g^-1^, 7.58 to 9.90 Log copies g^-1^ and 8.69 to 10.10 Log copies g^-1^, respectively. Overall, microbial evolution throughout SSF process is a much more dynamic process, and the quantities of total bacteria, LAB, *Bacillus* and fungi exhibited similar trends during the MTSSF and LTSSF processes.

### Microbial Composition and Dynamics by Illumina HiSeq Sequencing

Illumina HiSeq sequencing was used to investigate the microbial composition and dynamics during the MTSSF and LTSSF processes. After filtering the low-quality reads and chimeras, for bacterial communities, a total of 633,751 effective tags with an average length of 248 bp from all samples were obtained, and each sample contained 34,386 to 66,238 effective tags with different phylogenetic OTUs ranging from 175 to 485 were generated (Supplementary Table S1). For fungal communities, a total of 714,806 effective tags with an average length of 270 bp from all samples were obtained, and each sample contained 26,404 to 73,805 effective tags with different phylogenetic OTUs ranging from 32 to 134 were generated (Supplementary Table S2), via 97% sequence identity cutoff. Moreover, the rarefaction curves approached the saturation plateau (**Supplementary Figure S1**), which indicated that almost all bacterial and fungal communities could be well represented.

**FIGURE 4:**
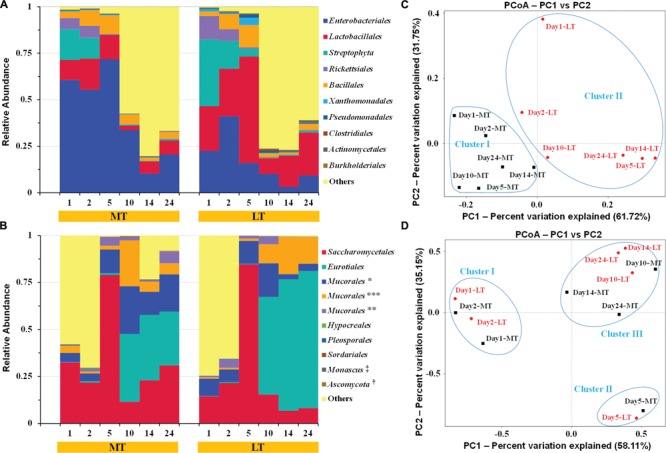
**Dynamics of relative abundances of the major bacterial **(A)** and fungal **(B)** orders, and the weighted unifrac distance PCoA of bacterial **(C)** and fungal **(D)** communities during the MTSSF and LTSSF processes, as obtained by Illumina HiSeq sequencing analysis.** The abundance was presented as of percentage of total effective bacterial sequences. The abundances of bacterial “other” orders were <0.40%. The abundances of fungal “other” orders were <0.20%. The taxonomy: ^∗^, IS–s-*Mucorales* sp.-*Mucorales*; ^∗∗^, IS–s-*Rhizomucor pusillus*-*Mucorales*; ^∗∗∗^, IS–s-*Rhizopus arrhizus* var. arrhizus-*Mucorales*; ^†^, Un–s-*Ascomycota* sp.; ^‡^, IS–s-*Monascus purpureu*. The percentages were the percentage of variation explained by the components.

In general, highly similar and successional dynamics of microbial communities were exhibited during the MTSSF and LTSSF processes at order level (**Figures 4A,B**) and genus level (**Supplementary Figure S2**). Groups of *Rickettsiales* and *Streptophyta* only dominated the initial 2 days but were retrieved at low frequencies (<1% of total sequences) until the end of SSF process (**Figure 4A**). Meanwhile, thermophilic *Eurotiales* first became prominent order on day 10 and predominated until the end of MTSSF and LTSSF processes. However, phylotypes of *Enterobacteriales, Lactobacillales, Bacillales, Saccharomycetales*, and *Mucorales* both prevailed the whole MTSSF and LTSSF processes (**Figures 4A,B**). Among these prevailing orders, despite some variation at some moment (**Figures 4A,B**), (i) the relative abundance of *Lactobacillales* and *Saccharomycetales* first increased from days 1 to 5, then decreased from days 5 to 10, and finally increased from days 10 to 24; (iii) the relative abundance of *Enterobacteriales* and *Bacillales* first increased from days 1 to 5, then decreased from days 5 to 14, and finally increased from days 14 to 24; (iii) the relative abundance of *Mucorales* first increased from days 1 to 10, then decreased from days 10 to 14, and finally increased from days 14 to 24. Nevertheless, several facts should be highlighted (**Figures 4A,B**): (i) the relative abundance of *Enterobacteriales* in MTSSF process constituted from 10.30% to 71.73%, while obviously lower in LTSSF process and the percentages ranged from 3.16 to 41.06%; (ii) the relative abundance of *Lactobacillales* in MTSSF process constituted from 2.34 to 16.68%, while obviously higher in LTSSF process and the percentages ranged from 8.43 to 57.39%; (iii) the relative abundance of *Eurotiales* in MTSSF process decreased steadily from 36.10 to 28.63% on days 10 to 24, while obviously higher in LTSSF process and the percentages gradual increased from 52.00 to 72.97% on days 10 to 24.

Variations in some small proportions of the top 10 bacterial groups of *Xanthomonadales* (0.10 to 3.94%), *Pseudomonadales* (0.17 to 1.85%), *Clostridiales* (0.02 to 0.98%), *Actinomycetales* (0.03 to 0.68%), and *Burkholderiales* (0.03 to 0.57%), and fungal groups of Hypocreales (0.00 to 0.42%), *Pleosporales* (0.00 to 0.41%), *Sordariales* (0.00 to 0.25%), and *Ascomycota* sp. (0.01 to 0.10%) were observed during the MTSSF and LTSSF processes. Other bacterial orders of *Rhizobiales* (0.01 to 0.14%), *Rhodobacterales* (0.00 to 0.29%), *Bacteroidales* (0.03 to 0.22%), *Flavobacteriales* (0.00 to 0.17%), *iii1–15* (0.00 to 0.16%), *Desulfobacterales* (0.00 to 0.16%), *Rhodospirillales* (0.00 to 0.14%), and *Campylobacterales* (0.00 to 0.14%), and other fungal orders of *Capnodiales* (0.00 to 0.17%), *Pezizales* (0.00 to 0.07%), and *Mortierellales* (0.00 to 0.05%) were also observed during the MTSSF and LTSSF processes.

### Statistical Analysis of Microbial Diversity and Richness

Alpha diversity indexes were conducted to evaluate the microbial richness and diversity varied during the MTSSF and LTSSF processes (Supplementary Tables S1 and S2). For bacterial richness and diversity (Supplementary Table S1), the observed OTUs indexes during the MTSSF process generally declined from 261 to 148, in contrast, the observed OTUs indexes during the LTSSF process markedly increased from 147 to 407 during the LTSSF process. Opposite evolution patterns of Chao 1 and ACE values were also observed. However, for fungal richness and diversity (Supplementary Table S2), similar evolution patterns of alpha diversity indexes were observed during the MTSSF and LTSSF processes, for example, the observed OTUs indexes decreased during the MTSSF and LTSSF processes from days 1 to 10 and days 1 to 5, and afterward increased. Notably, obviously lower in bacterial alpha diversity indexes of Shannon, observed OTUs, Chao 1, and ACE indexes during the MTSSF process compare with LTSSF process were observed after days 2, 5, and 10, respectively.

**FIGURE 5:**
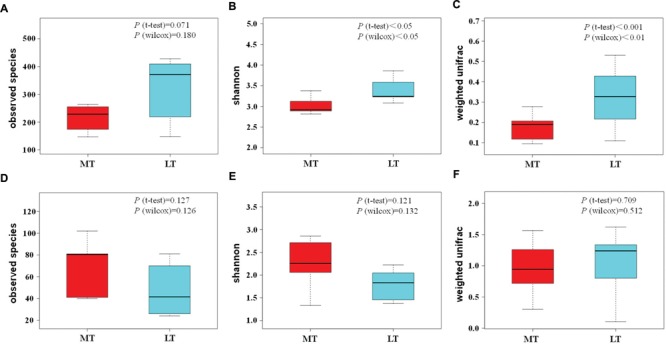
**Box and whisker plots of the variance in observed species **(A)**, shannon **(B)**, beta diversity based on weighted unifrac **(C)** of bacterial communities, and variance in observed species **(D)**, shannon **(E)**, beta diversity based on weighted unifrac **(F)** of fungal communities, as obtained by Illumina HiSeq sequencing analysis, respectively**.

In addition, the box and whisker plots showed that obviously lower (*P* > 0.05) and significantly lower (*P* < 0.05) in bacterial alpha diversity indexes of observed OTUs and Shannon indexes were observed during the MTSSF process compare with the LTSSF process, respectively (**Figures 5A,B**). However, commonly higher in fungal alpha diversity indexes were shown during the MTSSF process compare with LTSSF process. Furthermore, the box and whisker plots showed that dramatically higher (*P* > 0.05) in fungal alpha diversity indexes of observed OTUs and Shannon indexes were observed during the MTSSF process compare with the LTSSF process (**Figures 5A,B**).

Principal coordinate analysis was conducted to evaluate the similarities in microbial communities using the weighted UniFrac distance during the MTSSF and LTSSF processes. For bacterial communities, samples from MTSSF process and LTSSF process tended to form clusters (**Figure 4C**), the weighted UniFrac distance in MTSSF process were significantly lower (*P* < 0.01) than that in LTSSF process (**Figure 5C**). However, for fungal communities, clusters separately tended to form days 1 and 2, days 5, and days 10, 14, 24 both during the MTSSF and LTSSF processes (**Figure 4D**), no significant difference in the weighted UniFrac distance in the MTSSF and LTSSF processes (**Figure 5F**). Moreover, both ANOSIM and MRPP tests indicated that obviously differences (*R* > 0, *P* > 0.05) in bacterial communities but highly similarities (*R* < 0, *P* > 0.05) in fungal communities were exhibited in the MTSSF and LTSSF processes (**Table 1**).

**Table 1:** Analysis of similarities (ANOSIM) and MRPP to test for differences in microbial communities of MTSSF and LTSSF processes.

MT vs. LT	ANOSIM		MRPP	
	*R*	*P*	*R*	*P*
16S	0.076	0.174	0.019	0.215
ITS	-0.096	0.761	-0.029	0.714

*R < 0 means non-significantly differences among the two batches, *P* > 0.05 means statistically non-significant.*

### Relationships between Environmental Variables and Microbial Communities

**FIGURE 6:**
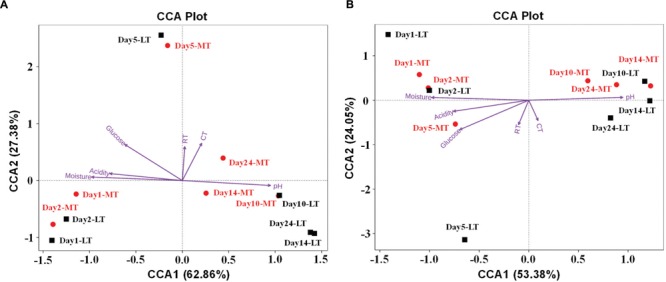
**Canonical correspondence analysis (CCA) of bacterial **(A)** and fungal **(B)** community structure as obtained by Illumina HiSeq sequencing and measurable environmental variables.** Arrows indicate the direction and magnitude of environmental parameters associated with microbial community structure. RT, room temperature. CT, core temperature.

The effect of environmental factors on the bacterial (**Figure 6A**) and fungal (**Figure 6B**) communities distribution was evaluated by CCA. Overall, the two axes explained 90.24 and 77.43% of the variation in bacterial and fungal community differentiation, respectively, suggesting the remarkable correlation between microbial structure and environmental factors. Moisture and acidity showed strongly positively correlation with bacterial and fungal composition at the mesophilic stages, but negative correlation with bacterial and fungal composition at cooling and maturation stages (**Figure 6**). However, core temperature (pile temperature) and pH showed negative correlation with bacterial and fungal composition at the mesophilic stages, but positively correlation with bacterial and fungal composition at cooling and maturation stages (**Figure 6**). Furthermore, Daqu samples from days 5 were distributed between days 1 to 2 and days 10 to 24 (**Figure 6**). Pearson’s correlation analysis revealed that moisture, acidity and glucose were mainly positively correlated with the relative abundances of *Enterobacteriales, Lactobacillales, Streptophyta, Rickettsiales, Bacillales*, and *Saccharomycetales* but negatively correlated with *Eurotiales* and *Mucorales* (Supplementary Table S3). However, pH showed contrasting correlations (Supplementary Table S3). Temperature were mainly positively correlated with the relative abundances of *Enterobacteriales, Lactobacillales, Bacillales, Saccharomycetales*, and *Mucorales* but negatively correlated with *Streptophyta, Rickettsiales*, and *Eurotiales* (Supplementary Table S3).

## Discussion

Traditional Chinese Daqu starters contain highly complex microbial communities, the microbial composition correlates with environmental parameters prevailing during the SSF process. Temperature, moisture, pH, and acidity are traditionally considered the crucial environmental variables used to evaluate Daqu SSF process. Nowadays, increasing attention is likely to be poured to the microbial composition of Daqu correlates with environmental factors prevailing during the fermentation process. This study combined molecular techniques to reveal microbiota dynamics during the MTSSF and LTSSF processes of Daqu starters differing in temperature regimes with consideration for potential relation to environmental variables.

As expected, the thermal profile was followed the quintessential evolution of Daqu SSF process, the temperature quickly rose to thermophilic temperature at thermophilic stages of MTSSF and LTSSF processes (**Figure 1A**), these might derived from heat generation due to the degradation of sugars and proteins by microbial activity (Troy et al., 2012). However, a noteworthy increase of reducing sugar contents was observed at phase A of the thermophilic stage during the LTSSF process (**Figure 1B**), this fact highlighted the intense microbial decomposition activity and the even faster decomposition raw materials to reducing sugar than the utilization of reducing sugar. Similar tends have also been observed by previously reported (Sulaiman et al., 2014). Proteases activity was appropriate indicator of decomposition of organic matter. In this study, the proteases activities and amino acid nitrogen contents slightly increased at the mesophilic stages, and significantly increased at thermophilic stages of MTSSF and LTSSF processes. Furthermore, pH first decreased at the mesophilic stages, and thereafter markedly increased at thermophilic stages of MTSSF and LTSSF processes (**Figure 1C**). This first decline in pH was attributed to the production of organic acids from the breakdown of sugars by the microbes (Liu et al., 2011), and the thereafter increase in pH was attributed to the decomposition of organic acids and to the production of amino acids and peptides fractions associated with protein degradation in the raw materials (Awasthi et al., 2014). Nevertheless, (i) in case of MT process relatively higher temperature profile was observed than LT process at the mesophilic, thermophilic and cooling stages (**Figure 1A**); (ii) significantly (*P* < 0.05) higher in pH and lower in titratable acidity were displayed at the mesophilic and thermophilic stages of the MTSSF process compare with the LTSSF process (**Figure 1C**); (iii) significantly (*P* < 0.05) higher in protease activity and amino acid nitrogen content were displayed on day 14 during MTSSF process compare with the LTSSF process (**Figure 2A**); all these facts demonstrated that the MTSSF process had a higher degradation rates of organic matter than LTSSF process at the mesophilic, thermophilic, and cooling stages, which were coincided with previously reported (Awasthi et al., 2015).

Daqu starters are primary liquefying and saccharifying agents that are used to initiate fermentation in the production of Chinese liquor and vinegar. Amylase and glucoamylase, the main mediators of SSF process, are the major contributors to the liquefaction and saccharification abilities. The amylase activity increased rapidly from days 2 to 5, however, the glucoamylase activity obviously declined throughout the SSF process (**Figure 2**), these can be attributed to the fact that the thermal stability of amylase, which was much better than glucoamylase at high incubation temperature of approximately 60°C (Soni et al., 2003). Nevertheless, significantly (*P* < 0.05) higher in glucoamylase activity were noted after the mesophilic stage of the MTSSF process compare with the LTSSF process (**Figure 2**), which was also indicated with its higher temperature profile and degradation rates of organic matter at the mesophilic, thermophilic and cooling stages (**Figure 1**), these results further suggested that the microbial activity in MTSSF process was higher comparatively to the LTSSF process.

Generally, most microorganisms are supposed to disappear and the microbial population was obviously declined when temperature reaches the thermophilic range (Chroni et al., 2009). Consequently, microbial quantities decreased significantly (*P* < 0.05) at phase A of thermophilic stages during the SSF process (**Figure 3**). Nevertheless, their quantities increased significantly (*P* < 0.05) at phase B of thermophilic stages, which suggested that some microbes showed the ability to survive as thermotolerant and an intensive thermophilic recolonization could proceed at phase B of thermophilic stages during the SSF process (**Figure 3**). However, higher levels of total bacteria, LAB, *Bacillus*, and fungi were displayed at the thermophilic stages of the MTSSF process compare with the LTSSF process, this might conditioned by higher temperature and lower titratable acidity during the MTSSF process compare with the LTSSF process. Production of enzyme depends on microbial biomass. Thus, this could provide a reason that the microbial activity in MTSSF process was higher comparatively to the LTSSF process. The microbial quantities showed decreased and increased trends at cooling stages during MTSSF and LTSSF processes, respectively. Theses contradictory tendencies might provide a direct evidence that persistent high temperature might result as deleterious at cooling stage (Insam and De Bertoldi, 2007), in consideration of higher in temperature but no obviously difference in moisture, pH and acidity in MTSSF process than that in LTSSF process (**Figure 1**).

In general, highly similar and successional dynamics of microbial communities were exhibited during the MTSSF and LTSSF processes at order level (**Figures 4A,B**). Phylotypes of *Enterobacteriales, Lactobacillales, Bacillales, Saccharomycetales*, and *Mucorales* both predominated the whole MTSSF and LTSSF processes, these members possessed a remarkable capacity to adapt to a wide range of temperatures and moisture levels and were widespread in various Daqu starters and other high-temperature ecosystems (Mouchacca, 2007; Adams et al., 2010; Wang and Xu, 2015). However, groups of *Rickettsiales* and *Streptophyta* only dominated the mesophilic stages, these groups were reported as obligate endosymbionts, and low water availability and high temperature could have a deleterious effect on these communities (Botella et al., 2010; Warner et al., 2010). Meanwhile, thermophilic *Eurotiales* first became prominent order on day 10 and dominated until the end of MTSSF and LTSSF processes. These microbiota dynamics coincided with Fen-Daqu fermentation process (Zheng et al., 2014) and our previously reports (Li et al., 2015), and can be explained by “systematic robustness” principle (Brenner et al., 2008; Freedman and Zak, 2015).

Interestingly, the relative abundance of *Enterobacteriales* in MTSSF process was obviously higher comparatively to the LTSSF process, but the relative abundance of *Lactobacillales* and *Eurotiales* in MTSSF process was obviously lower comparatively to the LTSSF process (**Figure 4B**). These contradictory discrepancies could be due to: (i) the restriction from the higher temperature and lower titratable acidity during the MTSSF process compare with the LTSSF process (**Figure 6**, Supplementary Table S3), for example, the growth of major genus of *Thermoascus* within *Eurotiales* (**Supplementary Figure S2**) were restrained when the temperature was >50°C (Kalogeris et al., 2003); (ii) the difference in initial relative abundance originated from the non-autoclaved raw materials and the open and uncontrolled industrial production environment (Zheng et al., 2012). CCA and Pearson’s correlation analyses further clarified that the lower in acidity correlated with the lower in relative abundance of *Lactobacillales* in MTSSF process (Zheng et al., 2014), and higher in temperature correlated with lower but higher in relative abundance of *Eurotiales* and *Enterobacteriales* in MTSSF process, respectively (**Figures 4** and **6**; Supplementary Table S3). These results might provide a direct evidence that persistent high temperature at cooling stage might have a deleterious effect on *Eurotiales* communities, in consideration of higher in temperature but no obviously difference in moisture, pH and acidity in MTSSF process than that in LTSSF process (**Figure 1**).

Moreover, CCA results revealed that moisture and acidity were the most important factors influencing bacterial and fungal composition at the mesophilic stages (**Figure 6**), however, core temperature (pile temperature) and pH were the most important factors influencing bacterial and fungal composition at cooling and maturation stages (**Figure 6**). In addition, CCA results indicated that Daqu samples from days 5 were distributed between days1 to 2 and days 10 to 24 (**Figure 6**), revealing that microbial structure transition happened at thermophilic stages under environmental stress of moisture, pH, acidity and pile temperature, significant microbial composition transition was also reported in fermentation pit mud of Chinese strong-flavored liquor (Tao et al., 2014; Hu et al., 2016). Meanwhile, our results suggested that lower bacterial richness and diversity but higher fungal richness and diversity were observed during the MTSSF process compare with the LTSSF process (**Figure 5**). Similarly, both PCoA, ANOSIM and MRPP analyses indicated that obviously differences (*R* > 0, *P* > 0.05) in bacterial communities but highly similarities (*R* < 0, *P* > 0.05) in fungal communities were exhibited in the MTSSF and LTSSF processes (**Figure 4**; **Table 1**). These results indicated that bacterial community and diversity was likely to be more sensitive to environmental variables adjustments than fungal community and diversity. Therefore, it could be more appropriate to consider that a significant proportion of microorganisms growing at thermophilic stages during the SSF process were actually thermotolerant and drought-resistant community, and the microbial communities can adapt to variations in environmental conditions by changes in the thermotolerant and drought-resistant community structure during the SSF process.

## Conclusion

There was considerable consistency of the microbial composition during the MTSSF and LTSSF processes. The microbial communities can adapt to variations in environmental conditions by changes in the thermotolerant and drought-resistant community structure during the SSF process. However, different environmental variables affect microbial composition during the MTSSF and LTSSF processes. The microbial activity in MTSSF process was higher comparatively to the LTSSF process. Obviously differences (*R* > 0, *P* > 0.05) in bacterial communities but highly similarities (*R* < 0, *P* > 0.05) in fungal communities were exhibited in the MTSSF and LTSSF processes. These profound understanding might help to effectively control the traditional Daqu SSF process by adjusting relevant environmental parameters.

## Author Contributions

Conceived and designed the experiments: PL, LL. Performed the experiments: PL, XL, XW. Generated and analyzed the data: PL, WL, LL. Wrote the paper: PL.

## Conflict of Interest Statement

The authors declare that the research was conducted in the absence of any commercial or financial relationships that could be construed as a potential conflict of interest.

## Supplementary Material

FIGURE S1 **Rarefaction curves based on the OTUs at the cutoff of 97% 16S rRNA **(A)** and ITS1 regions **(B)** of fungal rRNA genes sequence similarity obtained by Illumina HiSeq Sequencing**.

FIGURE S2 **Dynamics of relative abundances of the major bacterial **(A)** and fungal **(B)** genus during the MTSSF and LTSSF processes, as obtained by Illumina HiSeq sequencing analysis.** The abundance was presented as of percentage of total effective bacterial sequences. The abundances of bacterial “other” genera were <0.40%. The abundances of fungal “other” genera were <0.20%. The taxonomy: ^∗^, IS–s-*Mucorales* sp.-*Mucorales*; ^††^, Un–s-*Hypocreales* sp.
