# Supplementary material for: Environmental Factors Affecting Microbiota Dynamics during Traditional Solid-state Fermentation of Chinese Daqu Starter
Source: Front Microbiol. 2016 Aug 4;7:1237. doi: 10.3389/fmicb.2016.01237 (PMC4972817; doi:10.3389/fmicb.2016.01237)
Supplement: Supplementary file 1 [file Table_1.PDF]

Supplementary Table S1 Observed 16S rRNA Illumina MiSeq sequencing results and alpha diversity indices in samples.

Samples	No. of								Alpha diversity (97%)					
	Raw Tags	Clean Tags	Effective Tags	Base (nt)	Q20 (%)	Q30 (%)	GC%	Effective %	Observed species	Shannon	Simpson	Chao1	ACE	Goods coverage
Day1-MT	65,317	64,612	63,713	16097978	99.23	98.49	55.22	95.08	261	3.137	0.807	374.1	452.005	0.997
Day1-LT	66,622	64,656	63,129	16149353	99.08	98.22	54.37	92.30	147	3.073	0.805	186.417	191.606	0.999
Day2- MT	54,536	53,979	53,115	13444977	99.28	98.59	54.21	95.62	253	2.922	0.745	411.2	430.827	0.997
Day2-LT	40,975	40,506	39,622	10009581	99.22	98.49	53.49	94.09	224	3.248	0.815	268.634	283.34	0.998
Day5-MT	59,735	59,190	58,261	14724832	99.28	98.59	54.28	96.08	236	2.817	0.744	402.733	446.529	0.996
Day5-LT	35,714	35,340	34,386	8700060	99.26	98.55	51.21	94.54	378	3.588	0.798	392.575	413.521	0.998
Day10-MT	52,479	52,120	51,834	12649256	99.29	98.68	46.4	97.30	170	2.891	0.797	241.323	255.632	0.998
Day10-LT	59,377	58,990	58,384	13962917	99.3	98.72	43.09	96.23	351	3.242	0.787	486.284	547.334	0.996
Day14-MT	54,991	54,578	54,124	13123125	99.27	98.69	42.8	95.93	229	2.925	0.77	331.098	356.501	0.997
Day14-LT	54,157	53,695	53,260	12987697	99.27	98.65	43.28	96.54	401	3.251	0.819	515.725	559.215	0.996
Day24-MT	67,108	66,569	66,238	16151718	99.27	98.67	44.79	97.25	148	3.379	0.851	245.235	235.732	0.998
Day24-LT	38,647	38,320	37,685	9262580	99.29	98.68	45.33	95.58	407	3.864	0.875	454.53	473.117	0.997

Q20, 99% accuracy of effective tags; Q30, 99.9% accuracy of effective tags; GC, GC content of effective tags.
