# Supplementary material for: Environmental Factors Affecting Microbiota Dynamics during Traditional Solid-state Fermentation of Chinese Daqu Starter
Source: Front Microbiol. 2016 Aug 4;7:1237. doi: 10.3389/fmicb.2016.01237 (PMC4972817; doi:10.3389/fmicb.2016.01237)
Supplement: Supplementary file 2 [file Table_2.PDF]

Supplementary Table S2 Observed ITS1 Illumina MiSeq sequencing results and alpha diversity indices in samples.

Samples	No. of								Alpha diversity (97%)					
	Raw Tags	Clean Tags	Effective Tags	Base (nt)	Q20 (%)	Q30 (%)	GC%	Effective %	Observed species	Shannon	Simpson	Chao1	ACE	Goods coverage
Day1-MT	70,196	68,922	68,844	19241272	99.18	98.34	47.61	96.41	78	2.169	0.661	101.333	95.184	0.999
Day1-LT	75,392	73,847	73,805	20907569	99.09	98.14	51.64	96.24	79	1.959	0.565	96.1	93.141	0.999
Day2-MT	74,080	72,736	72,675	20336482	99.1	98.15	50.6	96.28	80	2.078	0.583	89.714	92.576	0.999
Day2-LT	63,262	62,161	62,129	17408192	99.15	98.26	49.75	96.66	70	2.244	0.651	76.5	78.751	1.000
Day5-MT	68,932	65,169	65,049	18084501	99.25	98.53	34.57	92.17	48	1.333	0.409	69.857	72.083	0.999
Day5-LT	65,066	63,865	63,824	17669102	99.24	98.51	34.33	96.45	23	1.375	0.438	26.75	32.198	1.000
Day10-MT	57,436	56,283	56,170	14888372	99.19	98.42	47.9	95.61	40	2.355	0.755	46	46.869	1.000
Day10-LT	71,481	70,217	70,142	18274580	99.21	98.45	51.24	96.59	29	2.047	0.674	33.2	37.302	1.000
Day14-MT	73,560	72,182	72,088	19298585	99.18	98.37	50.91	95.95	106	2.862	0.805	153.571	150.007	0.999
Day14-LT	30,849	29,647	29,596	7363519	99.21	98.46	56.85	94.35	32	1.458	0.484	33.2	34.619	1.000
Day24-MT	60,741	54,223	54,080	14550699	99.24	98.52	46.01	87.31	77	2.714	0.795	80.5	84.172	0.999
Day24-LT	29,850	26,524	26,404	6583928	99.26	98.54	57.01	87.19	53	1.719	0.526	59.6	63.932	1.000

Q20, 99% accuracy of effective tags; Q30, 99.9% accuracy of effective tags; GC, GC content of effective tags.
