# Supplementary material for: Environmental Factors Affecting Microbiota Dynamics during Traditional Solid-state Fermentation of Chinese Daqu Starter
Source: Front Microbiol. 2016 Aug 4;7:1237. doi: 10.3389/fmicb.2016.01237 (PMC4972817; doi:10.3389/fmicb.2016.01237)
Supplement: Supplementary file 3 [file Table_3.PDF]

**Supplementary Table S3** Pearson's correlation coefficients between environmental measurable variables and dominant bacterial and fungal orders.

| | **pH** | **Acidity** | **Glucose** | **Moisture** | **CT** | **RT** |
|---|---|---|---|---|---|---|
| ***Enterobacteriales*** | -0.567 | 0.341 | 0.451 | 0.657* | 0.205 | 0.31 |
| ***Lactobacillales*** | -0.48 | 0.609* | 0.696* | 0.355 | 0.032 | 0.215 |
| ***Streptophyta*** | -0.700* | 0.503 | 0.229 | 0.766** | -0.514 | -0.414 |
| ***Rickettsiales*** | -0.759** | 0.583* | 0.225 | 0.800** | -0.48 | -0.35 |
| ***Bacillales*** | -0.615* | 0.678* | 0.727** | 0.482 | 0.557 | 0.747** |
| ***Saccharomycetales*** | -0.293 | 0.292 | 0.719** | 0.285 | 0.446 | 0.459 |
| ***Eurotiales*** | 0.835** | -0.680* | -0.671* | -0.851** | -0.106 | -0.228 |
| ***Mucorales*** | 0.669* | -0.617* | -0.671* | -0.704* | 0.446 | 0.245 |

** Correlation is significant at $p<0.01$. * Significant at $p<0.05$. RT, room temperature. CT, core temperature.
