# Supplementary figures and images for: Environmental Factors Affecting Microbiota Dynamics during Traditional Solid-state Fermentation of Chinese Daqu Starter
Source: Front Microbiol. 2016 Aug 4;7:1237. doi: 10.3389/fmicb.2016.01237 (PMC4972817; doi:10.3389/fmicb.2016.01237)

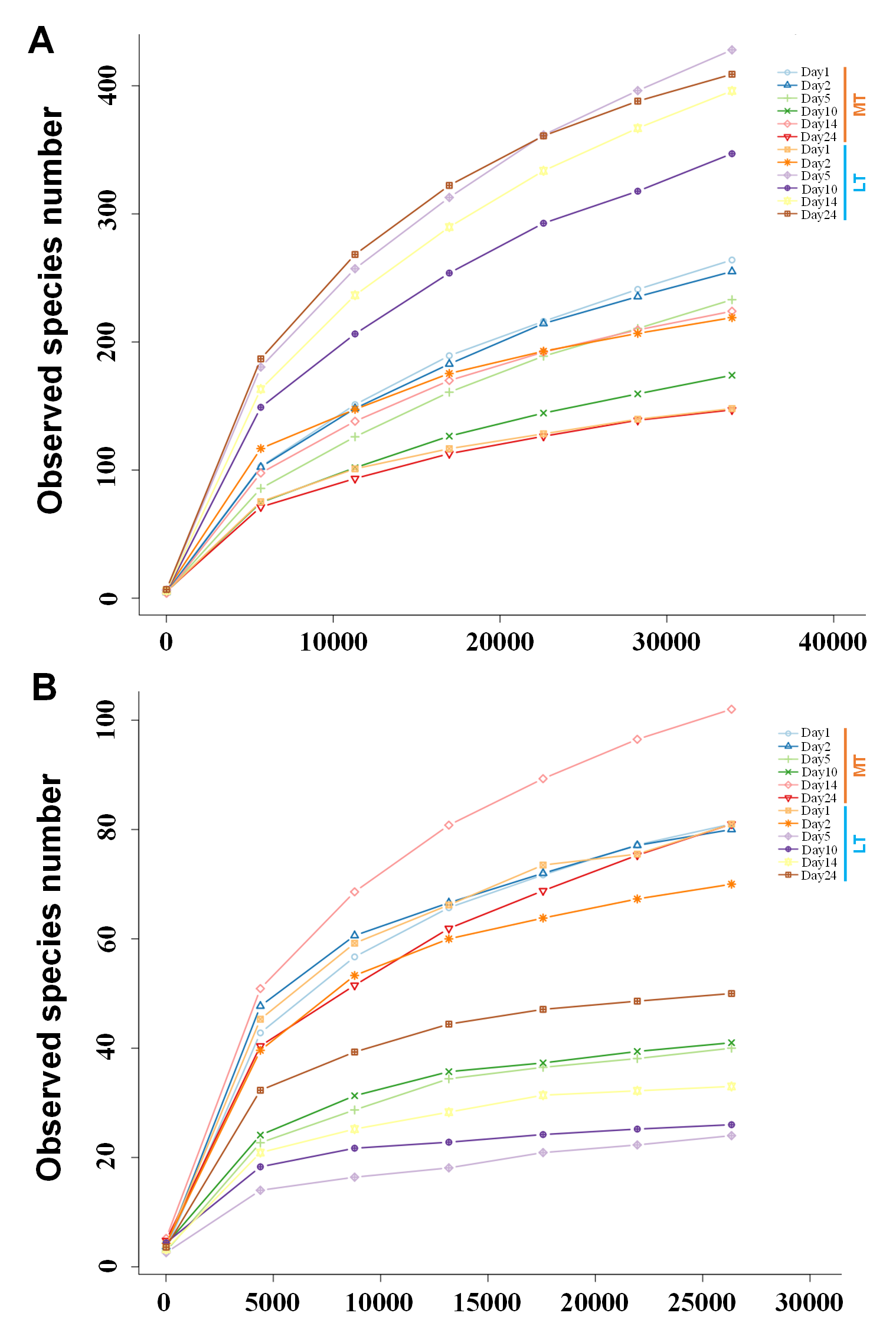

Supplement: FIGURE S1 — Rarefaction curves based on the OTUs at the cutoff of 97% 16S rRNA (A) and ITS1 regions (B) of fungal rRNA genes sequence similarity obtained by Illumina HiSeq Sequencing. [file Image_1.TIF]

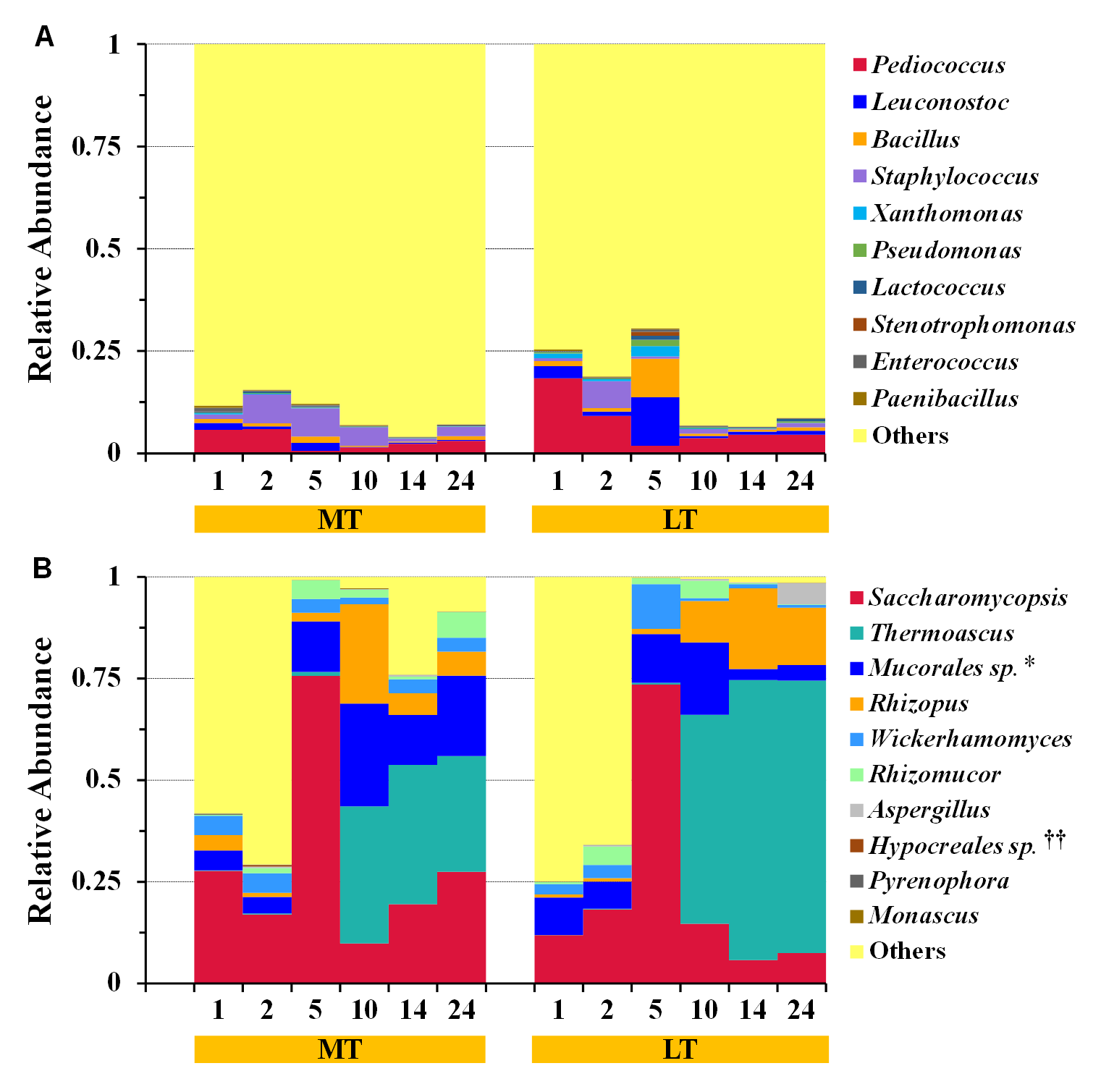

Supplement: FIGURE S2 — Dynamics of relative abundances of the major bacterial (A) and fungal (B) genus during the MTSSF and LTSSF processes, as obtained by Illumina HiSeq sequencing analysis. The abundance was presented as of percentage of total effective bacterial sequences. The abundances of bacterial “other” genera were <0.40%. The abundances of fungal “other” genera were <0.20%. The taxonomy: ∗, IS–s-Mucorales sp.-Mucorales; ††, Un–s-Hypocreales sp. [file Image_2.TIF]
